# Polysaccharide Hydrogels as Delivery Platforms for Natural Bioactive Molecules: From Tissue Regeneration to Infection Control

**DOI:** 10.3390/gels11030198

**Published:** 2025-03-12

**Authors:** Fabrizia Sepe, Anna Valentino, Loredana Marcolongo, Orsolina Petillo, Anna Calarco, Sabrina Margarucci, Gianfranco Peluso, Raffaele Conte

**Affiliations:** 1 Research Institute on Terrestrial Ecosystems (IRET), National Research Council of Italy (CNR), Via Pietro Castellino 111, 80131 Naples, Italy; fabriziasepe@cnr.it (F.S.); anna.valentino@cnr.it (A.V.); loredana.marcolongo@cnr.it (L.M.); orsolina.petillo@cnr.it (O.P.); sabrina.margarucci@cnr.it (S.M.); gianfranco.peluso@unicamillus.org (G.P.); raffaele-conte@cnr.it (R.C.); 2 National Biodiversity Future Center (NBFC), 90133 Palermo, Italy; 3 Faculty of Medicine and Surgery, Saint Camillus International University of Health Sciences, Via di Sant’Alessandro 8, 00131 Rome, Italy

**Keywords:** natural polysaccharides, bioactive compounds, biocompatibility, sustainable materials, drug delivery systems, biodegradability

## Abstract

Polysaccharide-based hydrogels have emerged as indispensable materials in tissue engineering and wound healing, offering a unique combination of biocompatibility, biodegradability, and structural versatility. Indeed, their three-dimensional polymeric network and high water content closely resemble the natural extracellular matrix, creating a microenvironment for cell growth, differentiation, and tissue regeneration. Moreover, their intrinsic biodegradability, tunable chemical structure, non-toxicity, and minimal immunogenicity make them optimal candidates for prolonged drug delivery systems. Notwithstanding numerous advantages, these polysaccharide-based hydrogels are confronted with setbacks such as variability in material qualities depending on their source, susceptibility to microbial contamination, unregulated water absorption, inadequate mechanical strength, and unpredictable degradation patterns which limit their efficacy in real-world applications. This review summarizes recent advancements in the application of polysaccharide-based hydrogels, including cellulose, starch, pectin, zein, dextran, pullulan and hyaluronic acid as innovative solutions in wound healing, drug delivery, tissue engineering, and regenerative medicine. Future research should concentrate on optimizing hydrogel formulations to enhance their effectiveness in regenerative medicine and antimicrobial therapy.

## 1. Introduction

**Figure 1:**
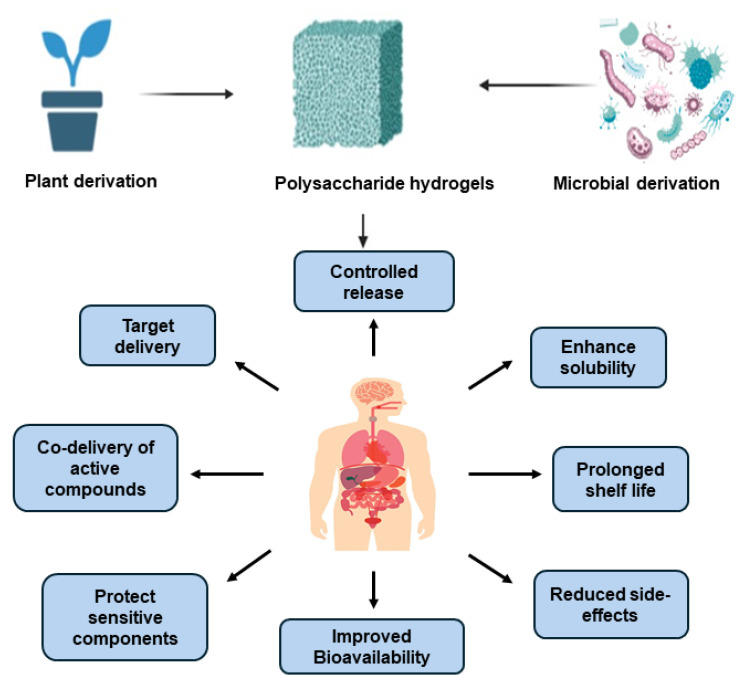
Advantages of plant and microbial derived polysaccharide hydrogels. Image created with BioRender.com.

With the rising demand for biodegradable and non-toxic drug delivery systems, polysaccharide-based hydrogels have emerged as a leading alternative to synthetic polymer carriers, offering enhanced biocompatibility and controlled release mechanisms. These hydrogels play a crucial role in biomedical applications, particularly in drug delivery, wound healing, and antimicrobial therapies, where their ability to encapsulate and regulate the release of bioactive compounds improves therapeutic outcomes while minimizing systemic side effects [1,2]. Additionally, their renewable and biodegradable nature aligns with the growing emphasis to waste management and demonstrating the potential for enhanced and enduring sustainability [3,4]. Polysaccharide-based hydrogels are derived from natural sources, including plant-based polysaccharides such as starch, pectin, cellulose or plant proteins such as and zein, as well as those produced through enzymatic activity, such as dextran, pullulan, and hyaluronic acid. These materials exhibit unique physicochemical properties—high water absorption, biocompatibility, and tunable mechanical strength—that make them ideal candidates for biomedical applications (Figure 1) [5,6]. Their structural and mechanical properties can be tailored through cross-linking strategies, swelling behavior modulation, and responsiveness to external stimuli, enabling precise control over drug release kinetics and therapeutic effectiveness [7,8]. Beyond drug delivery, polysaccharide-based hydrogels have demonstrated significant potential in tissue engineering and regenerative medicine. Their ability to retain moisture, encapsulate bioactive agents, and provide a favorable microenvironment for cell growth enhances wound healing and infection control while effectively modulating inflammatory responses [9]. Comparative studies indicate that these hydrogels exhibit lower environmental impact than conventional synthetic polymers, making them promising candidates for eco-friendly biomedical solutions [10]. This review explores the biomedical applications of various polysaccharide-based hydrogels, with a particular focus on their advantages in drug delivery, wound healing, and infection control. Additionally, we discuss current challenges, including scalability and mechanical limitations, and highlight future research directions aimed at optimizing their performance in biomedical and regenerative medicine applications.

### Outline of the Review

This review explores the biomedical applications of plant- and microbial-derived polysaccharide-based hydrogels, focusing on drug delivery, wound healing, and regenerative medicine. Section 2 outlines the key properties that enhance their suitability for bioactive release. Section 3 and Section 4 examine hydrogels from plant- and microbial-derived polysaccharides and proteins, respectively. Section 5 discusses emerging trends, including nanocomposites and smart hydrogels, and future directions for clinical and industrial development.

## 2. Polysaccharide Hydrogel System Characteristics

Polysaccharide-based hydrogels obtained from natural sources have gained significant interest due to their inherent biocompatibility, biodegradability, and versatility across various fields. Composed of long-chain carbohydrate polymers such as cellulose, hyaluronic acid, and starch derivatives, these hydrogels exhibit a remarkable capacity to retain water while maintaining their structural framework. Their natural origin provides key advantages, including reduced toxicity, minimal ecological footprint, and favorable interactions with biological environments [11]. Consequently, these hydrogels have been extensively explored in biomedical applications such as drug delivery, wound care, and tissue regeneration [12]. Their ability to form hydrogels via physical or chemical cross-linking enables broad adaptability, ranging from scaffolds supporting cellular growth to carriers for controlled therapeutic delivery. However, the successful incorporation of bioactive molecules requires mild cross-linking strategies to preserve molecular integrity and functionality. The cross-linking approaches for polysaccharide-based hydrogels are primarily categorized into physical and chemical methods [13]. Physically cross-linked hydrogels are assembled through non-covalent interactions, forming reversible networks that do not require chemical cross-linking agents. This characteristic allows for mild gelation conditions, beneficial for preserving biomolecular structure and activity [14]. Several mechanisms contribute to physical cross-linking, including electrostatic interactions, hydrophobic associations, ionic binding with multivalent ions, van der Waals forces, and host-guest inclusion complexes [15]. In contrast, chemically cross-linked hydrogels rely on irreversible covalent bonds, often facilitated by reactive small molecules such as monomers, photo-initiated groups, or oligomers [16]. Functional groups like carboxyl and amine moieties present in polysaccharide chains are instrumental in covalent polymerization. By incorporating reactive groups such as thiols, acrylates, or alkenes, polysaccharides can undergo targeted chemical modifications to enable stable cross-linking [17]. Both physically and chemically cross-linked hydrogels incorporate bioactive agents through weak interactions, entrapment, or cleavable linkages. These weak forces allow for a tunable approach to loading and releasing bioactive compounds, making polysaccharide-based hydrogels highly adaptable for diverse therapeutic applications. The ability to facilitate controlled release through reversible adsorption or stimulus-induced cleavage ensures precise regulation over biomolecule delivery kinetics [18]. Notably, the integration of hydrogels into biomolecule delivery represents a major advancement, as their intrinsic properties significantly influence drug release dynamics. Due to their biocompatible nature, these hydrogels seamlessly interact with biological systems without inducing adverse effects. Their high water retention capacity mimics the extracellular matrix, promoting biomolecule diffusion and facilitating cellular functions [19]. Furthermore, hydrogel properties can be tailored to optimize specific parameters such as mechanical strength, swelling characteristics, and degradation rates, thereby ensuring efficiency in controlled drug delivery [20]. Their capability to encapsulate various molecules, including proteins, peptides, and nucleic acids, further enhances their utility [21]. Additionally, hydrogels offer protection against enzymatic degradation and immune clearance, prolonging the circulation of therapeutic agents within the body [22]. Some hydrogels also exhibit stimuli-responsive behaviors, enabling the on-demand release of bioactive agents under specific environmental triggers. This feature allows for highly regulated biomolecule delivery, responding to changes in external stimuli such as ionic strength, light exposure, pH variation, magnetic or electric fields, temperature fluctuations, redox potential, or the presence of specific biomolecules [23]. Different strategies are employed for biomolecule release, including diffusion-based delivery, where hydrogel porosity dictates release kinetics, and swelling-controlled release, where hydrogel restructuring modulates molecule dispersion [24]. Biodegradation-mediated delivery leverages the natural breakdown of hydrogels, often via enzymatic activity, to gradually release therapeutic agents [25]. Affinity-based mechanisms further enable modulated release by leveraging interactions between hydrogel components and encapsulated biomolecules, ensuring sustained therapeutic effects based on molecular affinity parameters [26]. Lastly, link-breaking strategies involve the covalent attachment of biomolecules, with controlled cleavage under physiological conditions to achieve precise, time-dependent release [27].

## 3. Plant Derived Polysaccharides

**Figure 2:**
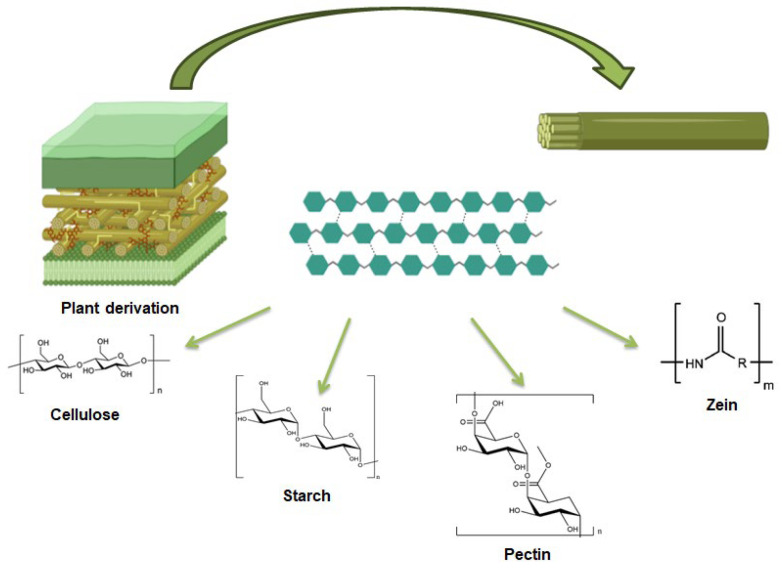
Plant-derived polysaccharides and proteins. Image created with BioRender.com.

Plant-derived polysaccharides and proteins represent a significant source for developing innovative biomaterials with diverse medical applications, offering both practical and economic advantages [28], as already discussed for polysaccharides of marine origin in Sepe et al. [23]. Numerous studies have highlighted the potential of these polysaccharides for enhancing the delivery of therapeutic agents, such as antioxidants, anti-inflammatory compounds, and anticancer drugs, by improving their stability and bioavailability within biological systems [28]. Their unique properties allow for the formulation of controlled-release drug delivery systems, which can protect active ingredients during transit through the body and enable their gradual and targeted release at specific sites, making them valuable for applications including immunotherapy. Additionally, plant-derived polysaccharides are employed in the creation of biomaterials such as hydrogels, scaffolds, and membranes, which are utilized in tissue engineering, wound care, and regenerative medicine [29]. These polysaccharides are also used in the food industry as natural thickeners, stabilizers, and biodegradable packaging materials [30], as well as in environmental applications for water purification and soil conditioning [31]. Figure 2 displays the structures of plant derived polysaccharides and proteins.

### 3.1. Starch

#### 3.1.1. Starch: Origin and Property

Starch is a natural polysaccharide that serves as an energy reserve in both plants and animals. It is predominantly stored in seeds, tubers, roots, and stem pith of plants (e.g., maize, wheat, potatoes, and tapioca). Industrially, starch is primarily extracted from cereal grains, tubers or the pith of plants [32]. Starch possesses a range of properties that make it valuable across various industries. Its strong hydrophilicity gives it a high affinity for water, making it easily soluble in aqueous solutions. This is crucial for its use as a thickening agent in food products and as a binder in pharmaceutical formulations. The viscosity of starch solutions can vary depending on concentration, temperature, and type, allowing for versatility in producing gels, pastes, and other formulations where viscosity control is essential [33]. When heated in water, starch granules undergo gelatinization, resulting in gel formation that provides texture, stability, and consistency in food products [34]. After gelatinization, starch gels can undergo retrogradation upon cooling, leading to a firmer gel or crystallization, which helps control food texture and prevent staling. Starch also can form flexible, transparent films when dispersed in water and dried. This property is used to produce biodegradable packaging and coatings for food and pharmaceutical products [35]. The size of starch granules is key to their function. Rice starch with small granules is highly valued in medical and cosmetic powders for topical use, effectively absorbing skin oils and acting as an excipient in dry shampoos and as a lubricant in surgical materials. Starch-based excipients enhance drug formulation and production by improving safety, cost-effectiveness, and product quality, serving as binders, diluents, disintegrants, lubricants, and glidants in granules, capsules, and tablets [35].

#### 3.1.2. Starch-Based Hydrogels Applications

Starch-based hydrogels form three-dimensional networks created by crosslinking of starch linear molecules [36]. In their manufacture, starch molecules are cross-linked by physical, chemical or enzymatic processes to form a network capable of absorbing and retaining large amounts of water. Starch-based hydrogels have diverse applications across various fields, including biomedicine, agriculture, food, and environmental engineering. In particular, such hydrogels have been investigated as carriers for encapsulating bioactive natural compounds such as plant extracts, essential oils and phytochemicals with therapeutic properties. These compounds find application in wound healing, anti-inflammatory processes and as antimicrobial therapies. The hydrogel support facilitates the controlled release of the molecules, extending their benefits in therapeutic treatments. For example, Ghaffar et al. [37] developed a hydrogel consisting of poly(amide/acrylic acid) to promote the release of rutin, a flavonoid glycoside found in citrus fruits with anti-inflammatory and anti-allergic properties. In vitro studies revealed a pH-dependent release behavior of rutin from the hydrogel. Moreover, in vivo experiments showed that the hydrogel effectively inhibited inflammation of colon by reducing toxicity. This suggests that such pH-sensitive hydrogels could serve as an efficient anti-inflammatory approach to enhance the therapeutic efficacy of rutin in treating inflammatory conditions. Stable hydrogels were successfully formed by D’Aniello et al. [38] in the presence of the green tea extract and glycerol in the starch suspension. The controlled and steady release of bioactive compounds from the hydrogel matrix over time indicated that small molecules, such as polyphenols, interacted effectively with the rice starch hydrogel network [38]. Phosphorylated starch, using sodium tripolyphosphate as a crosslinking agent, has demonstrated a high encapsulation efficiency for anthocyanins from purple maize, achieving a productivity rate of 87.63% [39]. Additionally, starch modified with octenyl succinic anhydride (OSA) in its amorphous form is currently being utilized for the encapsulation of hydrophobic polyphenols [40]. The OSA modified waxy maize starch was able to interact with tea polyphenols leading to the gradual release of catechins during in vivo digestion [40]. All these described examples are summarized in Table 1.

### 3.2. Pectin

#### 3.2.1. Pectin: Origin and Property

Pectin is a polysaccharide found in the cell walls of plants, particularly in fruits. Extracted commercially from various citrus sources such as apple, pomace, and oranges, pectin undergoes extraction under mildly acidic conditions. It is primarily categorized into two major groups: high methoxyl pectin and low methoxyl pectin [41]. As a high-value functional food ingredient, pectin finds widespread application as a gelling agent and stabilizer in the food industry. Furthermore, its potential extends to biomedical realms, notably in targeted drug delivery and various biomedical applications. Rapid degradation by colonic microorganisms makes pectin a promising candidate for colon-targeted drug delivery systems [42].

#### 3.2.2. Pectin-Based Hydrogels Applications

Pectin-based formulations exhibit significant promises as innovative biomaterials for the development of implantable devices and prosthetics. In fact, it is biodegradable and suitable for use in implants, packaging materials and for the control of the viscosity of liquid products. Pectin biocompatibility is useful in biomedical applications, such as drug delivery systems and tissue engineering [43]. Pectin-based hydrogels have a remarkable capacity to absorb and retain significant amounts of water. Their soft and rubbery consistency minimizes irritation to surrounding tissues, rendering them exceptional candidates for applications in artificial skin and tissue engineering [44]. Additionally, pectin-based hydrogels find diverse utility as matrices for controlled drug delivery, soft contact lenses, protein separation, and cell encapsulation [45]. Nevertheless, pectin formulations suffer from drawbacks such as premature drug release, low mechanical strength, suboptimal drug loading efficiency, and limited shear stability. Consequently, complexes with other polysaccharides were developed. For example, Mishra et al. produced pH sensitive polyacrylamide grafted pectin by simple graft copolymerization. This method showed a better ability to gel and form a film than the pectin [46]. These complexes are particularly adapt to deliver natural substances [46]. A hydrogel mixture of pectin/polyethylene glycol and calcium carbonate microparticles was synthesized for the targeted release of bovine serum albumin (BSA) into the colon. The encapsulation efficiency of BSA in the hydrogel blend was about 98%. In vitro studies demonstrated that the hydrogels released the protein for approximately 9 h in the colon, highlighting their ability to transport drugs [47]. Yang et al. [48] developed caseinate-reinforced pectin hydrogel beads (PCHG-CAS) with the aim of improving the stability of (-)-epigallocatechin (EGC) and preventing its degradation. PCHG-CAS formed a compact structure due to the hydrogen bond between caseinate and pectin, achieving a high encapsulation efficiency of EGC of 93.39%. EGC showed a rapid release from PCHG-CAS in the first 30 min, and between 30 and 120 min showed a stabilization phase, reaching 76% of release. The addition of protein demonstrated their effect on release kinetics, significantly slowing the release of EGC from PCHG-CAS into water. This condition simultaneously ensured a controlled release of the hydrogel under simulated gastrointestinal conditions. Notably, EGC in PCHG-CAS remained chemically stable for a storage period of 6 days at 37 °C, preventing epimerization, oxidation, dimerization and trimerization [48]. Bostancı et al. [49] studied photo-cross-linked pectin/gelatin hydrogels for curcumin release. Hydrogels of different compositions were successfully synthesized from methacrylate derivatives of gelatin and pectin (PCMA). The hydrogel consisting of the highest PCMA content had pores with an average diameter of 44 μm, demonstrating remarkable stability, retaining 38% of its initial weight after 21 days in phosphate-buffered saline (PBS). In PBS (10 mM) at pH 7.4, curcumin was released four times faster than at pH 5.0. Furthermore, disk diffusion assays demonstrated the efficacy of the hydrogels against *S. aureus* and *E. coli* bacteria. In addition, tests of the hydrogels with L929 fibroblasts using Alamar Blue assays showed their cytocompatibility [49]. Hydrogel beads with varying ratios of alginate and pectin were investigated by Wu et al. [50] to encapsulate Pickering emulsions containing resveratrol. Cross-linking the beads with Ca^2^⁺ allowed better flexibility in drug delivery. Alginate/pectin hydrogel beads exhibited pH reactivity when tested under simulated gastrointestinal conditions. This limited the release of resveratrol into the simulated stomach-like gastric fluid and promoted greater release into the simulated intestinal fluid (SIF), thereby improving the bioaccessibility of resveratrol [50]. Mala and Anal [51] explored the release and protection capacity of bromelain encapsulated within hydrogel beads made of pectin and maize starch acid-resistant. The use of the 4.5:1.5 (*w*/*w*) pectin/starch ratio allowed to reach 81.25% of the encapsulation efficiency. In fact, the addition of a starch resistant to the acid improves the entrapment of bromelain, with a better swelling, a greater stability at gastric level and the prolongation of the release compared to the hydrogels constituted only by pectin. Bromelain encapsulated in hydrogels showed faster release in simulating intestinal fluid (pH 7.4) than gastric fluid (pH 1.2) [51]. Alsakhawy et al. [52] developed a hydrogel using Arabic gum (AG) and pectin to encapsulate naringenin (NAR). The developed NAR-loaded hydrogel achieved an impressive encapsulation efficiency of approximately 99.79% with a load of drug of about 16%. The NAR-loaded AG-pectin hydrogel accelerated wound healing by promoting angiogenesis and collagen deposition. Furthermore, the mRNA expression of inflammatory (TNF-α) and apoptosis-related (BAX) markers was significantly reduced [52]. The water solubility was improved by making a combination of quaternized chitosan (QCS) and pectin which also improved the antibacterial properties, the tensile strength as well as stability of the hydrogel. Incorporation of propolis into the film improved the wound healing property. The propolis-loaded hydrogel films exhibited antioxidant activity ranging from 22% to 37%. Release percentages of propolis were approximately 22% for QCS-loaded films, 78% for pectin films, and 64% for QCS–pectin hydrogel films. Wound closure with propolis-loaded QCS hydrogel films was around 3.25%, but pectin addition improved wound closures up to 53.13%, also showed substantial antibacterial activity against *S. aureus* and *S. pyogenes*. Additionally, the hydrogel films were non-toxic to NCTC clone 929 mouse fibroblast cells [53]. The cited examples are summarized in Table 2.

### 3.3. Cellulose

#### 3.3.1. Cellulose: Origin and Property

Cellulose is a polysaccharide consisting of linear chains of glucose molecules linked together through β(1→4) glycosidic bonds. It is the most abundant organic compound on Earth and serves as a fundamental structural component in the cell walls of plants, providing rigidity and strength. It is synthesized by plants through the process of photosynthesis, utilizing carbon dioxide and water to produce glucose, which is then polymerized into cellulose [54]. Cellulose has strong and rigid fibers; it is insoluble in water due to the extensive hydrogen bonding between its glucose chains but is able to absorb and retain moisture. This property is beneficial in various applications, including as a thickening agent in food products and as a component in wound dressings. Moreover, cellulose is chemically stable under normal conditions, resisting degradation from acids, alkalis, and most organic solvents [55]. The production of cellulose-based hydrogels typically involves the crosslinking of cellulose molecules using various methods such as physical, chemical or enzymatic crosslinking. These methods result in the formation of a network structure capable of absorbing and retaining large amounts of water [56].

#### 3.3.2. Cellulose-Based Hydrogels Applications

Cellulose-based hydrogels have numerous applications in a wide range of fields, including biomedical, agricultural, food and environmental engineering. In biomedicine, they can be used as wound dressings, scaffolds for tissue engineering, and drug delivery systems. They are also employed as components in diagnostic tools due to their soft and flexible nature, ability to mimic the extracellular matrix and to absorb water up to several thousand times their dry weight [57]. Cellulose-based hydrogels resulted particularly attractive for biomedical thanks to their biocompatibility as they offer a protective, aqueous environment that can shield sensitive cells and delicate drugs, including peptides and proteins. Hydrogels also facilitate efficient nutrient transport to cells and the removal of metabolic waste, can be easily modified with cell adhesion ligands, and may be injected in vivo as a liquid that solidifies at body temperature [58]. Phan et al. prepared cellulose hydrogel by physical cross-linking in a NaOH/urea medium with α-mangostin employed as an active pharmaceutical ingredient. The hydrogel’s inhibitory activity against the growth of MC3T3-E1 cells identifies it as a potential drug carrier for α-mangostin in ankylosing spondylitis treatment [59]. Gami et al. synthesized a xylan-β-cyclodextrin cellulose hydrogel through a chemical crosslinking method and investigated its absorption and release properties for curcumin. In phosphate-buffered saline (PBS), the drug on the surface of the hydrogel was rapidly released, resulting in a faster initial release rate. Curcumin formed inclusion complexes with β-cyclodextrin within the gel, inhibiting its release [60]. A cellulose hydrogel matrix with pectin and mucin was synthesized using a urea/NaOH solvent system and cross-linked with epichlorohydrin to develop superabsorbent cellulose-based hydrogels [61]. The hydrogels were evaluated for their pH-responsive swelling behavior and in vitro drug release characteristics. Results indicated a significant increase in swelling capacity for the Pec/Muc-modified hydrogels compared to the unmodified cellulose hydrogel, with the highest swelling ratio reaching 2781% at pH 10 for the Pec-modified sample. Additionally, variations in drug release profiles were observed across different pH conditions. Hydrogels exhibiting greater swelling and reduced matrix erosion demonstrated a slower drug release due to an extended diffusion pathway [61]. Regenerated cellulose derived from sugarcane bagasse was utilized in the fabrication of hydrogels, employing epichlorohydrin as a crosslinking agent and green zinc oxide nanoparticles (ZNPs), synthesized using muskmelon seed extract. The ZNPs were characterized using field emission scanning electron microscopy and energy-dispersive X-ray analysis. The swelling behavior of the hydrogel was evaluated through swelling ratio measurements. For drug delivery applications, curcumin was selected as a model drug due to its notable antimicrobial and anticancer properties. Investigations such as Fourier transform infrared spectroscopy, X-ray diffraction, thermogravimetric analysis and scanning electron microscopy confirmed the incorporation of ZNPs and curcumin into the hybrid hydrogel. Optimization of drug loading efficiency was conducted using the Taguchi method. Furthermore, the antimicrobial efficacy of the curcumin-loaded hybrid hydrogel and pure cellulose hydrogel was assessed against *Staphylococcus aureus* and *Trichophyton rubrum*. These findings suggest that the developed biomass-derived hybrid hydrogel, loaded with curcumin, holds significant potential for applications in the treatment of skin infections [62]. Table 3 recaps the described applications.

### 3.4. Zein

#### 3.4.1. Zein: Origin and Property

Zein is a naturally occurring protein found in corn (maize) kernels. It is the major storage protein in corn and accounts for approximately 50% of the total protein content in maize. Zein is biocompatible, biodegradable and has excellent film-forming properties, allowing it to form thin, transparent films when dissolved in alcohol or other organic solvents. These films have barrier properties against moisture, oxygen, and other gases, making them useful for coating food and pharmaceutical products. Zein is insoluble in water, but soluble in alcohol and other organic solvents. This property allows for the production of water-resistant coatings and films with good thermal stability and suitable for applications that require heat processing, such as extrusion coating and hot-melt adhesives [63]. Finally, Zein is gluten-free and suitable for individuals with gluten sensitivities or celiac disease. This property has led to its use in gluten-free food products as a substitute for wheat-based ingredients [63].

#### 3.4.2. Zein-Based Hydrogels Applications

The adaptability of zein-based hydrogels allows the integration of a wide range of drugs, peptides and small molecules. In tissue engineering, plant-based hydrogels play a critical role as a supportive scaffold to promote cell growth and tissue regeneration. Their porous architecture facilitates the diffusion of nutrients and oxygen, thereby promoting vital cellular activities. In addition, these hydrogels can be customized to reproduce the mechanical characteristics of tissues, creating the conditions for cells to attach, proliferate and differentiate. Zein-based hydrogels are largely diffused also for drug delivery application. For example, Ji et al. [64] encapsulated cinnamaldehyde (CA) in a composite carriers made of zein and chitosan, taking advantage of its antimicrobial properties. The interaction between CA and the zein–chitosan composite significantly reduced the volatilization rate of CA. The mass ratio of zein to CS resulted in varying structural configurations, affecting encapsulation efficiency, release behavior, and antimicrobial efficacy. Interestingly, the co-carrier’s presence slowed the release of CA [64]. Interestingly, systems made of zein loaded with hydrophobic substances were also studied. For instance, zein devices loaded with maytansine not only improved tumor targeting in non-small-cell lung cancer (A549 cells), but also reduced possible side effects due to maytansine [65]. Zein hydrogels with curcumin demonstrated significant antibacterial activity, with increasing inhibition observed as curcumin content rose [66]. Encapsulation of curcumin in whey protein isolate (WPI)-zein composite nanogels significantly improved its dispersibility [67]. Additionally, zein-hyaluronic acid composite nanogels, prepared using a layer-by-layer assembly technique, effectively enhanced the light, thermal, and storage stability of both curcumin and quercetagetin [68]. Furthermore, numerous studies have reported the encapsulation quercetin [69], resveratrol [70], rutin [71], β-carotene [72], vitamin D3 [73] and retinol [74] as hydrophobic agents in zein vectors. Finally, curcumin-loaded zein-hyaluronic acid gels demonstrated strong anti-cancer activity against CT26 colorectal cancer cells, probably due to the hyaluronic acid enhancing CD44 receptor targeting [75]. The cited applications were summarized in Table 4.

## 4. Microbial-Derived Polysaccharide

**Figure 3:**
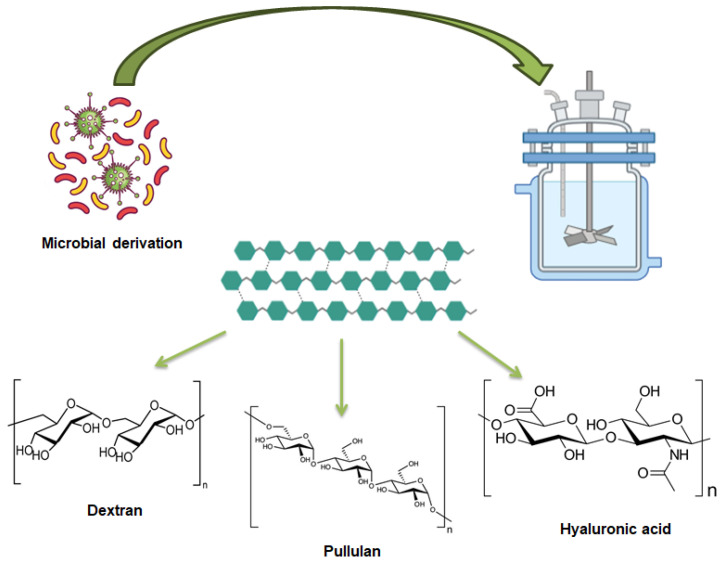
Microbial-derived polysaccharides. Image created with BioRender.com.

Microbial derived polysaccharides represent a promising and versatile category of natural materials with substantial potential for various biomedical and industrial applications. Through targeted microbial growth, these polysaccharides can be tailored to possess specific properties, enhancing their effectiveness as carriers for therapeutic agents [76,77]. Microbial (bacterial and fungal) sources are increasingly favored for their ability to produce high-yield polysaccharides. Different studies have demonstrated that polysaccharides from microbial sources can improve the stability, bioavailability, and targeted delivery of bioactive compounds, such as antioxidants, anti-inflammatory agents, and anticancer drugs [78,79,80]. Their ability to form hydrogels, scaffolds, and membranes makes them invaluable in tissue engineering, wound care, and regenerative medicine, where they offer tailored support and controlled release of therapeutic agents. Beyond biomedicine, enzymatically derived polysaccharides are utilized in the food industry as natural additives and stabilizers, and in environmental applications for water treatment and pollutant removal [81]. Figure 3 displays the structures of microbial derived polysaccharides.

### 4.1. Dextran

#### 4.1.1. Dextran: Origin and Property

Dextran is nontoxic water-soluble branched homo polysaccharide of glucose linked via α-1, 6 glycosidic linkages with various branches at α (1, 2) or (1–4) and (1–6) positions [82] and with variable length. This polysaccharide is of microbial origin; in particular, it has been produced by lactic acid bacteria such as *Lactococcus*, *Carnobacterium*, *Weissella* and *Leuconostoc*, nonpathogenic species generally regarded as safe [83]. Dextran characteristic of water solubility, biodegradability, and biocompatibility make it a good candidate for the structuring of hydrogel-based platforms for drug delivery. Adequate chemical modifications allow obtaining dextran derivatives (dextran sulphate, thiolated dextran, phosphorylated dextran) improving drug administration. Different substituents can be linked to dextran to improve the performance of the material with high drug loading efficiency and targeting of specific cells [84]. In addition, dextran is a neutral polysaccharide and hydrogels, when used as a delivery vehicle, have the ability to cross the mucus layer due to their hydrophilicity [85]. Dextran hydrogels have the property of protecting drugs from enzymatic and chemical degradation prior to release. Due to the action of dextranase, the polysaccharides they contain can swell and dissolve rapidly [86].

#### 4.1.2. Dextran-Based Hydrogels Applications

The preparation process is critical to achieving important hydrogel properties such as mechanical strength, self-healing and swelling capacity [87]. There are several crosslinking methods used to prepare dextran-based hydrogels, which can be summarized as physical and chemical crosslinking or a combination of the two [85]. The ability of dextran to implement the hydrogel formulation compared to conventional dressings has been highlighted by several works. A hydrogel of halloysite nanotubes mixed with chitosan/oxidized dextran, showed strong antibacterial capacity, improving *Staphylococcus aureus*-infected resistant wounds [88]. Antimicrobial properties have been demonstrated in dextran/chitosan hydrogels loaded with polydopamine nanoparticles [89], and in an oxidizing dextran and gelatin hydrogel loaded with nanoparticles of cerium oxide and curcumin as bioactive compounds [90]. Amphiphilic alkylated dextran nanoparticles served to encapsulate curcumin, improving bioavailability and delivery to the desired site. The development of combined hydrogels based on dextran-hyaluronic acid loaded with Sanguinarine, derived from the roots of *Sanguinaria canadensis* L. and *Chelidonium majus* L., showed antibacterial activity against *S. aureus* and *Escherichia coli*. Long-lasting drug release and wound regeneration capacity were also demonstrated in a rat burn infection model [91]. In the field of skin tissue regeneration an injectable hydrogel composed of gelatin, oxidized dextran loaded with apocynin, a naturally occurring methoxy-substituted catechol isolated from the roots of *Apocynum cannabinum*, was stabilized by the dynamic Schiff base. The hydrogel had the characteristics of good injectability, self-healing and hemostatic properties. Moreover, studies on murine and human cell evidenced an acceleration in angiogenesis with wound inflammation reduction [92]. The specificity of rosmarinic acid, a natural polyphenol with a structure similar to dopamine, allowed the construction of a rosmarinic acid-grafted dextran/gelatin hydrogel [93]. The hydrogel loaded with the natural bioactive compound showed very rapid gelation (61.6 ± 2.8 s) and good mechanical properties. In addition to highlighting antibacterial properties, tests on rat models demonstrated the effectiveness of the gel in healing skin wounds. Dextran based cyclodextrins (DC) are often used for complexation [94]. These cyclodextrins consist of glucopyranoside units linked via α-1,4 glycosidic bonds, synthesized by bacterial enzymes (cyclodextrin glycosyltransferases). Due to their hydrophilic surface and hydrophobic cavity, they can form inclusion complexes with various lipophilic bioactive increasing their solubility, stability and bioavailability [95]. For example, hypromellose hydrogels with different DC was used to incorporate 3-O-Methylquercetin, a flavonoid from *Achyrocline satureioides* [95]. The formulation of the gel allowed the skin release of the bioactive molecule (100%), directing it to the site of replication of melanoma cells. DC-based hydrogels have numerous uses in the biomedical field. An example was the hydrogel composed of β-CD, polyethyleneimine (PEI) and silk fibroin cross-linked with epichlorohydrin and loaded with *Centella asiatica* extract and hydrocortisone acetate. The bioactive molecules were released effectively, even if the released *C. asiatica* extract was pH dependent, healing of bedsores was observed (in 6 days), which was not found in the negative control [96]. A cyclodextrin-based hydrogel loaded with gallic acid allowed a slow release of the phenolic acid (in 48 h) showing antibacterial activity against *S. epidermidis*, *S. aureus* and *K. pneumoniae* without causing any damage to the surrounding tissue [97]. Cui et al. [98] produced an hydrogel based on methyl-β-cyclodextrin and soluble soy polysaccharide loaded with *Satureja montana* L. essential oil. Compared with the soluble soybean hydrogel alone, the cyclodextrins gave a more compact gel structure. Furthermore, a good antibacterial activity of the hydrogel against *S. aureus* was verified in meat samples [98]. The described examples are summarized in Table 5.

### 4.2. Pullulan

#### 4.2.1. Pullulan: Origin and Property

Pullulan is a homopolysaccharide of natural origin, synthesized during the fermentation of the fungus *Aureobasidium pullulans* [99]. The polymer chemical structure is linear, consisting of maltotriose units interconnected by α-1,6 and α-1,4 glycosidic bonds that make the polysaccharides neutral and non-ionic [99]. Thanks to the regular alternation of glycosidic bonds, pullulan exhibits structural flexibility, good elastic properties, solubility in aqueous solvents and high thermal stability (melting temperature range 250–300 °C) [100]. Pullulan has the ability to form films and is also non-carcinogenic, non-ionic, non-irritant, non-hygroscopic, non-toxic, blood-compatible, biodegradable, hydrophilic, odorless and tasteless [101]. Its important properties make the polysaccharide applicable in a wide range of fields, from textiles to food packaging, cosmetics and pharmaceutical formulation, the limitation to the widespread use of pullulan concerns its production costs [100].

#### 4.2.2. Pullulan Based Hydrogels Applications

Pullulan has antibacterial, anticoagulant and anti-inflammatory properties and is therefore used as a carrier for trans-mucosal drug delivery systems and wound dressings, in capsule and tablet coatings or for topical formulation in the form of gels and oral films [102]. These unique characteristics are also exploited for the preparation of hydrogels for various applications [103]. Hydrogels characterized by a three-dimensional cross-linked structure are able to retain large volumes of water and swell without dissolving, trapping small biomolecules [104]. Bioactive compounds of natural origin, frequently used for the treatment of numerous pathologies, can be promisingly incorporated into pullulan hydrogels. In fact, the characteristics of the polymer allow significant quenching of free radicals, a useful function in skin wound treatment. An Asiatic traditional medicine for the treatment of inflammation and skin wounds, *Ulmus davidiana* var. japonica root bark, has been used for the development of pullulan hydrogel. Hydrogels containing *U. davidiana* had good thermal stability and mechanically improved properties compared to the pullulan-only gel film, with swelling capacity and skin adherence. Hydrogels containing up to 50% extract showed a cell viability of 90%. Furthermore, an improvement in wound healing was observed, collagen deposition with dermal regeneration was achieved in mouse wound model [105]. A pullulan-based hydrogel was formulated to load an aqueous extract of *Rhus verniciflua* a tree used in traditional Asiatic herbal medicine as immunostimulant and endowed with antioxidant, anticancer, anti-inflammatory and antimicrobial activities [106]. The pullulan hydrogel was able to release approximately 87% of the bioactive compound in 12 h of application, stabilizing the release in 24–48 h. The application of this hydrogel, capable of creating an anti-scratch film on the skin, in the treatment of atopic dermatitis highlighted a better infiltration of mast cells, thus confirming the therapeutic efficacy [106]. Curcumin is known for its antioxidant, antibacterial, and anti-inflammatory properties, but its low bioavailability and poor solubility in water limit its use as a drug. An injectable hydrogel platform of hyaluronic acid-pullulan and pluronic was designed for the slow release of curcumin to repair diabetic wounds [107]. In the hydrogel with optimized composition, 50% release of the bioactive was observed after 8 h, continuing the release over 24 h. Interestingly, using the curcumin-loaded hydrogel, a wound healing rate of 13 days was observed compared to the 35 days required without the bioactive compound [107]. Antibacterial, antifungal activities and bacteriostatic effect after 48 and 72 h against *S. aureus* and *E. coli*, respectively, was also found in a clove oil-loaded hydrogel synthesized by combining covalent and physical cross-linking methods [108]. The hydrogels loaded with optimized amounts of clove oil exhibited excellent mechanical properties, good swelling ability combined with rapid recovery and sustained release of oil, essential properties for wound dressing applications [108]. The potential of plant extracts released from pullulan hydrogels for wound dressings was investigated also by Pelin et al. [109]. Pullulan/poly(vinyl alcohol) hydrogels, prepared by an environmentally friendly method by covalent and physical cross-linking, were loaded with the hydroalcoholic extract of *Calendula officinalis* by dipping after loading. The formation of hydrogen bonds between the polymer and the bioactive allowed high loading efficiency. It was also possible to give the gel good mechanical properties, with bio adhesiveness improving as the amount of bioactive loaded increased. The pullulan-based hydrogel loaded with extracts of *C. officinalis* were also characterized by a high antioxidant and antibacterial activity, both against Gram-positive and Gram-negative bacteria, and were not cytotoxic against Human Dermal Fibroblasts cells [109]. The cited applications are summarized in Table 6.

### 4.3. Hyaluronic Acid

#### 4.3.1. Hyaluronic Acid: Origin and Property

Hyaluronic acid (HA) is a linear anionic polysaccharide that belongs to the glycosaminoglycan family, consisting of repeating units of N-acetyl-D-glucosamine and glucuronic acid [110]. Historically, HA has been obtained from rooster combs and bovine vitreous humor. However, microbial fermentation using streptococcus and bacillus species has become an alternative and widely adopted method for HA production due to its efficiency and reduced risk of contamination from animal-derived sources [111]. In the human body, HA is abundant in synovial fluid and vitreous humor, where it plays an important structural role in articular cartilage and skin [112]. HA possesses excellent water solubility and forms highly viscous solutions, contributing to its free radical scavenging activity, bacteriostatic properties, and facilitation of tissue repair [113]. However, the homopolymeric form of HA lacks sufficient mechanical strength and stability for use as a structural scaffold. To overcome this limitation, HA is chemically modified by cross-linking with ethyl esters, benzyl esters, or other biodegradable polymers, enhancing its mechanical properties while preserving biocompatibility [114]. These cross-linked HA hydrogels are highly adaptable and can be fabricated into various structures, including membranes, sponges, fibers, and scaffolds. They have been extensively explored for applications in wound healing, tracheal regeneration, and the repair of cartilage, vasculature, and neural tissues [114].

#### 4.3.2. Hyaluronan-Based Hydrogel Applications

HA-based hydrogels exhibit characteristics of biodegradability, great adaptability to environmental stimuli and the ability to stimulate the immune system. These characteristics make such hydrogels of great interest in biomedicine, in particular in tissue and regenerative engineering and drug administration [23]. For instance, Kwon et al. developed pH-responsive hydrogels incorporating the phenol isoliquiritigenin from licorice, employing hydroxyethylcellulose and HA for transcutaneous delivery. These hydrogels were obtained by conjugate Michael addition and allowed for improved drug release at pH above 7, indicating a pH dependence [115]. Furthermore, Xin Jin et al. designed a composite hydrogel consisting of tannic acid and carbon in dopamine-coated particles enriched with phenolic compounds. This hydrogel via oxidative coupling improved cross-linking and adhesion by establishing covalent and hydrogen bonds with wound tissues [116]. The hydrogel exhibited fast gelation speed (<6 s) and remarkable adhesiveness (>8.1 kPa). In vitro evaluations indicated minimal hemolysis activity, low cytotoxicity, and improved fibroblast proliferation and migration. An in vivo model of skin defect to which the hydrogel was applied demonstrated accelerated wound healing through photothermal treatment. It also reduced inflammation, alleviated tissue hypoxia and promoted angiogenesis and epithelialization [116]. In another study, Gallelli et al. [117] evaluated the therapeutic potential of HA nanohydrogels incorporated with quercetin and oleic acid. This formulation significantly reduced the healing time of skin wounds on the lower limbs of diabetic patients compared to free hyaluronic acid (0.2%) and exhibited a favorable safety profile with no adverse drug reactions [117]. Similarly, Conte et al. [118] formulated a novel periodontal drug delivery system by encapsulating *Opuntia ficus-indica* extract, recognized as anti-inflammatory, antioxidant and antibacterial, inside chitosan nanoparticles immersed in pluronic-hyaluronic hydrogels temperature responsive. This system demonstrated effective eradication of biofilms formed by *Streptococcus mutans*, *Pseudomonas aeruginosa*, and *Porphyromonas gingivalis* while disrupting extracellular polymeric substance formation. Additionally, these hydrogels exhibited immune-modulating properties [118]. In further research, the same authors developed HA-based hydrogels containing resveratrol-loaded chitosan nanoparticles for atopic dermatitis treatment.

The encapsulation in HA of the nanoparticles delayed their hydrolysis, allowing a controlled and sustained release of resveratrol. The formulation mitigated oxidative stress in human keratinocytes with TNF-α/INF-γ (HaCaT) and reduced the expression and secretion of pro-inflammatory cytokines [119]. The studies discussed in this section are summarized in Table 7.

## 5. Challenges and Future Directions

**Figure 4:**
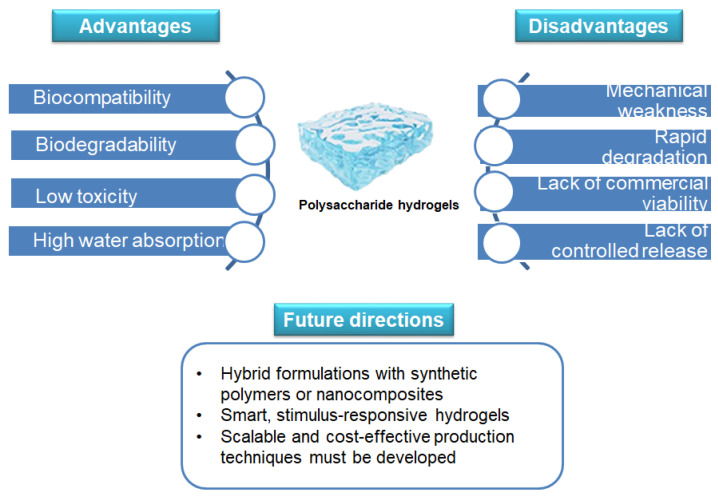
Advantages/disadvantages of natural polysaccharides. Image created with BioRender.com.

Despite their numerous advantages, polysaccharide-based hydrogels also present significant limitations that must be addressed to facilitate their widespread clinical and commercial adoption. One of the major challenges is their mechanical weakness, which can limit their use in load-bearing applications such as cartilage repair [120]. Many natural polysaccharides lack the necessary structural integrity and durability, making them prone to rapid degradation or mechanical failure under physiological conditions [121]. Furthermore, their high swelling capacity, while beneficial for bioactive release, can lead to instability in certain biomedical applications, reducing their functional lifespan [121]. Another limitation is the complexity of achieving precise control over degradation rates and drug release kinetics [122]. Polysaccharide-based hydrogels often exhibit variability in their degradation profiles due to differences in cross-linking methods, environmental conditions, and interactions with biological fluids. This inconsistency poses challenges for developing reproducible and standardized formulations suitable for regulatory approval [122]. Additionally, the potential for immunogenic responses or contamination from plant-derived or microbial sources needs careful evaluation to ensure patient safety [123]. To overcome these challenges, future research should focus on developing reinforced protein and polysaccharide-based hydrogels through hybrid formulations with synthetic polymers or nanocomposites. The incorporation of nanoparticles, graphene derivatives, or bioceramic fillers can significantly enhance mechanical properties, providing better structural stability for applications in tissue engineering and regenerative medicine [124]. Smart, stimulus-responsive hydrogels that react to changes in pH, temperature, or enzymatic activity can also improve control over bioactive release, ensuring more predictable therapeutic outcomes [125]. Issues related to large-scale production, cost-effectiveness, and reproducibility remain major hurdles in industrial manufacturing. Then, scalable and cost-effective production techniques must be developed to facilitate commercial viability [126]. Moreover, regulatory frameworks should also be adapted to accommodate the complexities of these materials, with standardized protocols for biocompatibility assessment, long-term stability testing, and large-scale production [127] (Figure 4).

## 6. Conclusions

Polysaccharide-based hydrogels represent a powerful and sustainable platform for the controlled release of natural bioactive compounds, with wide-ranging applications in drug delivery, wound healing, and antimicrobial therapies. Their biocompatibility, biodegradability, and tunable physicochemical properties make them ideal candidates for biomedical applications, allowing for precise modulation of drug release profiles and enhanced therapeutic outcomes. This review has explored hydrogels derived from plant-based polysaccharides and proteins, including starch, pectin, cellulose, and zein, as well as those obtained through microbial action, such as dextran, pullulan, and hyaluronic acid. The incorporation of these materials into hydrogel-based devices has demonstrated significant advantages, including improved stability, prolonged bioactive release, and enhanced interaction with biological tissues. The optimization of cross-linking mechanisms, swelling behavior, and responsiveness to external stimuli, permitted to tailor hydrogels for specific therapeutic needs, reducing side effects and increasing treatment efficacy. Moreover, the use of naturally derived bioactives within these hydrogels has shown promising results in accelerating wound healing, preventing infections, and modulating inflammatory responses, reinforcing their role in regenerative medicine and targeted drug delivery. As research continues to advance, further innovations in hydrogel formulation and functionalization are expected to drive the development of next-generation biomaterials that combine efficacy, sustainability, and biocompatibility.

## Figures and Tables

**Table 1:** Applications of starch-based hydrogels.

Application	Administration	Starch-Based Formulation	Benefits	References
Drug delivery	Oral route	Poly(starch/acrylic acid) (1:10 wt) hydrogel for rutin delivery	Inhibition of colonic inflammation with reduced toxicity	[37]
Drug delivery	Oral route	Glycerol-starch suspension with green tea extract	Steady and sustained release of these compounds over time	[38]
Drug delivery	Oral route	Phosphorylated starch with sodium tripolyphosphate as a crosslinking agent containing anthocyanins from purple maize	High encapsulation efficiency	[39]
Drug delivery	Oral route	Starch modified with octenyl succinic anhydride (OSA) containing tea polyphenols and catechins	Gradual release during digestion	[40]

**Table 2:** Applications of pectin-based hydrogels.

Application	Administration	Pectin-Based Formulation	Benefits	References
Drug delivery	Oral route	pH sensitive polyacrylamide grafted pectin to deliver natural substances	Better gelling and film forming ability than pectin	[36]
Colon localized release	Oral route	Calcium carbonate microparticles within a pectin/polyethylene glycol hydrogel blend for the targeted delivery of bovine serum albumin (BSA)	High encapsulation efficiency	[37]
Drug delivery	Oral route	Pectin hydrogel beads reinforced with caseinate (PCHG-CAS) to improve the stability of (−)-epigallocatechin (EGC)	Delayed EGC release in water and providing controlled release under simulated gastrointestinal conditions. EGC remained chemically stable over a 6-day storage period at 37 °C, preventing epimerization, oxidation, dimerization, and trimerization	[38]
Drug delivery	Oral route	Photo-crosslinked methacrylated derivatives of pectin/gelatin hydrogels containing curcumin	Stabilization of bioactive compounds	[49]
Drug delivery	Oral route	Hydrogel beads with varying alginate and pectin ratios to encapsulate Pickering emulsions loaded with resveratrol	pH-responsive behavior	[40]
Gastric delivery device	Oral route	Hydrogel beads made from pectin and acid-resistant maize starch containing bromelain	Better swelling properties, sustained release, and greater gastric stability compared to hydrogels made with pectin alone	[51]
Wound healing	Topical	Hydrogel with Arabic gum and pectin encapsulating naringenin	Acceleration of wound healing by promoting angiogenesis and collagen deposition and reduction in the mRNA expression of inflammatory markers and apoptosis-related factors	[52]
Wound healing	Topical	Quaternized chitosan and pectin hydrogel containing propolis	Strong antioxidant and antibacterial activity	[53]

**Table 3:** Applications of cellulose-based hydrogels.

Application	Administration	Cellulose-Based Formulation	Benefits	References
Treatment of ankylosing spondylitis	Scaffold	Cellulose hydrogel by physical cross-linking in a NaOH/urea medium with α-mangostin employed as an active pharmaceutical ingredient	Inhibitory activity against the growth of MC3T3-E1 cells	[59]
Drug delivery	Scaffold	Xylan-β-cyclodextrin cellulose hydrogel with curcumin	Curcumin forms inclusion complexes with β-cyclodextrin within the gel, prolonging the release	[60]
Drug delivery	Scaffold	Cellulose hydrogel matrix with pectin and mucin cross-linked with epichlorohydrin to develop superabsorbent cellulose-based hydrogels	Greater swelling and reduced matrix erosion; Slower drug release due to an extended diffusion pathway	[61]
Skin infection	Topical	Hydrogel of regenerated cellulose derived from sugarcane bagasse with zinc oxide nanoparticles delivering musk melon seed extract and curcumin	Strong antimicrobial efficacy	[62]

**Table 4:** Applications of zein-based hydrogels.

Application	Administration	Zein-Based Formulation	Benefits	References
Antimicrobial device	Oral route	Composite carrier made of zein and chitosan containing cinnamaldehyde	Interaction of drug and zein-chitosan composite reduces volatilization rate with good encapsulation efficiency, release behavior and antimicrobial efficacy	[64]
Anticancer delivery system	Oral route	Maytansine-loaded zein devices	Enhanced tumor targeting for non-small cell lung cancer (A549 cells) and reduced toxic side effects of maytansine	[55]
Drug delivery	Oral route	Curcumin-loaded zein devices	Enhanced antibacterial activity	[66]
Drug delivery	Oral route	Whey protein isolate (WPI)-zein composite nanogels containing curcumin	Improved dispersibility of Curcumin	[67]
Drug delivey	Oral route	Zein-hyaluronic acid composite nanogels containing curcumin and quercetagetin	Preparation using a layer-by-layer assembly technique; increase in the light, thermal, and storage stability of drugs	[68]
Carriers	Oral route	Zein hydrogel containing quercetin	Good encapsulation efficiency	[69]
Carriers	Oral route	Zein hydrogel containing resveratrol	Good encapsulation efficiency	[70]
Carriers	Oral route	Zein hydrogel containing rutin	Good encapsulation efficiency	[71]
Carriers	Oral route	Zein hydrogel containing β-carotene	Good encapsulation efficiency	[62]
Carriers	Oral route	Zein hydrogel containing Vitamin D3	Good encapsulation efficiency	[73]
Carriers	Oral route	Zein hydrogel containing retinol	Good encapsulation efficiency	[74]
Localized drug delivery to colorectal cells	Oral route	Curcumin-loaded zein–hyaluronic acid gel	Strong anticancer activity against CT26 colorectal cancer cells, attributed to the enhanced targeting of the CD44 receptor	[75]

**Table 5:** Applications of dextran-based hydrogels.

Application	Administration	Dextran-Based Formulation	Benefits	References
Antibacterial device for wound healing	Topical	Hydrogel of halloysite nanotubes mixed with chitosan/oxidized dextran.	Strong antibacterial capacity; Improvement of *S. aureus*-infected resistant wounds	[88]
Antimicrobial device for wound healing	Topical	Dextran/chitosan hydrogels loaded with polydopamine nanoparticles	Strong antimicrobial capacity	[89]
Antimicrobial device for wound healing	Topical	Oxidizing dextran and gelatin hydrogel, loaded with nanoparticles of cerium oxide and curcumin	Strong antimicrobial capacity	[90]
Wound healing	Topical	Combined hydrogels based on dextran-hyaluronic acid loaded with Sanguinarine	Antibacterial efficacy against *S. aureus* and *E. coli*; Long-duration drug release; Wound regeneration ability	[91]
Wound healing	Topical	Injectable hydrogel composed of gelatin and oxidized dextran loaded with apocynin	Good injectability, self-healing and hemostatic properties	[92]
Wound healing	Topical	Rosmarinic acid-grafted dextran/gelatin hydrogel	High biocompatibility, very rapid gelation times and good mechanical properties	[93]
Melanoma-directed delivery device	Topical	Hypromellose hydrogels with different dextran based cyclodextrins incorporating 3-O-Methylquercetin.	Improved skin release	[95]
Localized drug delivery	Oral route	Hydrogel composed of dextran β-CD, polyethylenimine and silk fibroin loaded with *Centella asiatica* extract and hydrocortisone acetate	pH dependent release	[96]
Localized drug delivery	Topical	Dextran cyclodextrin-based hydrogel loaded with gallic acid	Slow release; Antibacterial activity against *S. epidermidis*, *S. aureus* and *K. pneumoniae* without causing any damage to the surrounding tissue	[97]
Antibacterial device	Oral route (in association with ingested food)	Hydrogel based on dextran methyl-β-cyclodextrin and soluble soy polysaccharide loaded with *Satureja montana* L. essential oil.	Strong antibacterial action	[98]

**Table 6:** Applications of pullulan-based hydrogels.

Application	Administration	Pullulan-Based Formulation	Benefits	References
Wound healing	Topical	Pullulan hydrogel with *Ulmus davidiana* var. japonica root bark	Improved skin adherence	[105]
Anti-scratch film on the skin for the treatment of atopic dermatitis	Topical	Pullulan-based hydrogel loaded with an aqueous extract of *Rhus verniciflua*	Immunostimulant, antioxidant, anticancer, anti-inflammatory and antimicrobial	[106]
Repair diabetic wounds	Topical	Injectable hydrogel platform of hyaluronic acid-pullulan and pluronic containing curcumin	Controlled release of curcumin	[107]
Wound dressing	Topical	Clove oil-loaded pullulan hydrogel	Excellent mechanical properties, good swelling ability combined with rapid shape recovery; Sustained release of the oil	[108]
Wound healing	Topical	Pullulan/poly(vinyl alcohol) hydrogels loaded with the hydroalcoholic extract of *Calendula officinalis*	High loading efficiency; Good mechanical properties; Increased bioadhesiveness	[109]

**Table 7:** Applications of hyaluronic acid-based hydrogels.

Application	Administration	Hyaluronic Acid-Based Formulation	Benefits	References
Transdermal drug delivery	Topical	pH-sensitive hydrogel formed of hydroxyethyl cellulose and hyaluronic acid, containing isoliquiritigenin	pH-dependent drug release	[115]
Wound healing	Topical	Hyaluronic acid based composite hydrogel incorporating tannic acid and dopamine-coated carbon particles rich in phenols	Rapid gelation time; Good adhesive strength, Low hemolytic activity; Minimal cytotoxicity; Ability to promote fibroblast proliferation and migration	[116]
Treatment of lower limb skin wound in patients with diabetes mellitus	Topical	Hyaluronic acid-based nanohydrogel embedded with quercetin and oleic acid	Reduction in wound healing time without developing adverse drug reactions	[117]
Localized and sustained release in periodontal pockets	Scaffold	*Opuntia ficus-indica* extract encapsulated in chitosan nanoparticles embedded in pluronic–hyaluronic thermo-responsive hydrogel	System able to eradicate biofilms of *S. mutans*, *P. aeruginosa* and *P. gingivalis* and disrupt extracellular polymeric substance formation.	[118]
Treatment of atopic dermatitis	Topical	Hyaluronic acid hydrogel containing resveratrol-loaded chitosan (CS) nanoparticles	Delayed hydrolytic degradation; Decreased oxidative damage; Reduced secretion and gene expression of proinflammatory cytokines	[119]

## Data Availability

No new data were created or analyzed in this study.
